# Role of transcranial Doppler ultrasound in early assessment of acute post-traumatic brain injury

**DOI:** 10.1097/MD.0000000000045171

**Published:** 2025-10-17

**Authors:** Ahmed Mohamed Ekbal Ghoneim, Safaa Kamal Mohamed, Waleed Abdelhamid Hetta, Mona Gamalludin Alkaphoury

**Affiliations:** a Radio-Diagnosis Department, Faculty of Medicine, Ain Shams University, Cairo, Egypt.

**Keywords:** cerebral blood flow, CT brain score, secondary neurological deterioration, transcranial Doppler, traumatic brain injury

## Abstract

Traumatic brain injury (TBI) represents a significant global health and socioeconomic burden. This research aims to assess the accuracy of transcranial Doppler (TCD) ultrasound in the early assessment of cerebral blood flow following acute TBI and its utility as an early predictor of neurological outcomes, in correlation with computed tomography findings. A comparative study was conducted on 55 adult patients with acute TBI admitted to Helwan University Hospital between October 2021 and April 2023. TCD was performed within 8 hours post-trauma, and patients were monitored for secondary neurological deterioration (SND) at day 7 and for in-hospital mortality. Nineteen patients (34.5%) developed SND. These patients showed significantly higher admission Rotterdam and Marshall computed tomography scores (*P* < .001), lower end-diastolic velocity and mean flow velocities (*P* < .05), and higher pulsatility index (*P* < .001). Peak systolic velocity showed no significant difference. Receiver operating characteristic analysis identified pulsatility index and end-diastolic velocity as strong predictors of SND at day 7 post-trauma, with area under the curves of 0.923 and 0.884, sensitivity of 87.95% and 73.68%, specificity of 97.2% and 88.89%, and accuracy of 90.9% and 83.6%, respectively. A substantial proportion of TBI patients studied experienced SND within the initial 7 days following trauma. Early TCD assessment offers a valuable, noninvasive tool for identifying patients at risk, aiding in clinical triage and management.

## 1. Introduction

Traumatic brain injury (TBI) represents a significant worldwide healthcare and socioeconomic challenge. It impacts individuals across all age groups and is prevalent across both high- and low-resource communities.^[1]^ TBI can occur due to a variety of factors, such as, road traffic accidents (RTAs), falls from height (FFH), and physical assaults. The consequences can range from short-term complications to long-term disabilities and even death.^[2]^

Among the many scoring systems developed for trauma assessment, neurological evaluation, particularly through the use of coma scales, has been recognized as the most dependable indicator of prognosis in individuals with neurological injuries.^[3]^

The Glasgow Coma Scale (GCS) is a broadly accepted diagnostic approach employed to assess the consciousness level, clinical condition, and prognosis in TBI patients.^[4]^ The GCS categorizes over eighty percent of TBI patients presented to the emergency room (ER) as mild to moderate. However, secondary neurological deterioration (SND) occurs in an estimated 20% of cases during the initial post-traumatic week, despite early reassuring clinical presentations.^[5]^

Acute TBI consists of both primary and secondary injuries. Primary brain injury denotes the acute damage inflicted by the initial insult, directly affecting the brain parenchyma, whereas secondary brain injury evolves over time and is driven by various pathological processes, including neuronal and vascular damage, proteolytic cascades, oxidative stress, apoptosis, inflammation, and ischemia.^[6]^ Several clinical studies have linked poor outcomes in TBI to low cerebral blood flow (CBF) and ischemia, which may result from intracranial hypertension, systemic hypotension, cerebral edema, localized mass effect caused by hematomas, and disturbances in microvascular circulation.^[7]^

Computed tomography (CT) remains the standard diagnostic imaging tool employed in emergency care units for the evaluation of post-TBI. Although CT demonstrates an excellent negative predictive value, it is not a reliable predictor of subsequent neurological deterioration. Furthermore, the GCS, a key component of clinical assessment, lacks sensitivity in identifying high-risk patients who may experience neurological decline. Consequently, the need for an additional diagnostic tool to effectively screen this patient population is evident.^[8]^

Episodes of intracranial hypertension, which can be life-threatening, are commonly observed in patients with TBI. Prompt diagnosis and appropriate therapeutic interventions are critical, making intracranial pressure monitoring essential for effective clinical management.^[7]^

Currently, several advanced diagnostic imaging tools are available to assess CBF in the brain post-injury, including positron emission tomography, single-photon emission computed tomography, Xenon computed tomography (Xenon-CT), perfusion-weighted magnetic resonance imaging, and CT perfusion imaging. While these methods offer detailed insights into CBF, they are typically expensive and limited to a few specialized TBI centers, providing only transient snapshots of CBF dynamics.^[9]^

Transcranial Doppler ultrasonography (TCD) offers a sensitive, noninvasive modality for assessing cerebral blood flow in the intracranial arteries, where anatomical images are supplemented with physiological data.^[7]^ TCD uses pulsed-wave Doppler to detect vessels at varying depths and generate a spectral waveform, providing peak systolic velocity (PSV) and end-diastolic velocity (EDV) values.^[10]^ TCD represents one of the simplest approaches for vascular evaluation in clinical settings, being valued for patient comfort, ease of access, ability to perform successive assessments, and even continuous cerebral blood flow monitoring.^[11]^

TCD was successfully utilized in several studies to examine alterations in cerebral blood flow dynamics, particularly in the intensive care unit, as neurological impairment following TBI is often associated with cerebral ischemia. However, there are few studies using TCD in newly admitted TBI patients.^[12]^

While CT scans allow for early prediction of outcomes in cases presenting either absent or profound intracerebral damage, they are less effective in identifying patients with minor intracranial insults who may later experience SND.^[13]^ These patients can benefit from TCD, which can detect low diastolic blood flow velocity and high pulsatility index (PI) values indicative of high vascular resistance in cases with mild to moderate TBI (GCS 9–15) and normal or minor brain injuries detected on the initial CT scan.^[14]^

## 2. Objective of study

The objective of this research is to assess the accuracy of TCD for early evaluation of CBF following TBI and its role as an early predictor of neurological outcomes in these patients, in correlation with their CT findings.

### 2.1. Methodology

#### 2.1.1. Study design

Our study is a comparative, prospective analysis conducted on 55 adult patients (aged 17–55 years) with acute TBI who met the inclusion and exclusion criteria. These patients were presented at the Emergency care unit at Helwan University Hospital over an 18-month period, from October 2021 to April 2023. Participants were selected randomly.

#### 2.1.2. Ethical approval

The study was conducted following approval from the Research Ethics Committee of the Faculty of Medicine, Ain Shams University, with informed consent obtained from all participants.

### 2.2. Population

#### 2.2.1. Inclusion criteria

- Patients of both genders.
- Adult patients 17 years of age or older.
- Patients suffering from head trauma who presented to the ER within 8 hours post-injury.
- Patients who underwent TCD ultrasonography within 8 hours after head injury.
- Patients who received initial management and stabilization.

#### 2.2.2. Exclusion criteria

- Patients younger than 17 years old.
- Patients who suffered cardiac arrest before TCD examination.
- Patients who exhibited hemodynamic instability after initial resuscitation and required vasopressors and/or mechanical ventilation at the time of examination.
- Patients with open head injuries or those requiring urgent surgical intervention.
- Patients with contraindications for CT imaging (e.g., pregnancy).
- Patients with inadequate ultrasound windows or any craniotemporal lesions impeding a satisfactory TCD examination (e.g., temporal bone fractures or transcalvarial brain herniation).

## 3. Methods

Upon admission, all enrolled individuals underwent an initial clinical assessment, which included a comprehensive history (mechanism and time of injury) and a physical examination, with particular attention to vital signs and GCS scores.

### 3.1. TCD ultrasonography

TCD ultrasonography was performed in the ER within the first 8 hours post-injury, following the protocol outlined below:

- The ultrasound machine used was the **TOSHIBA APLIO 400**.
- A skilled radiologist conducted the examination using an **S probe** (3S phased array probe) with a 2 MHz transducer.
- The transducer was positioned anteriorly and slightly upward above the temporal region, directly in front of the tragus of the ear and immediately above the zygomatic arch (transtemporal window).^[15]^The probe angle and position were modified to obtain the clearest possible Doppler signal.
- Each middle cerebral artery (MCA) tracing was captured for a minimum of 10 cardiac cycles, with a duration of at least 30 seconds.^[8]^
- The following parameters were measured and recorded: PSV, EDV, and mean flow velocity (MFV) (in cm/s). Additionally, the PI was calculated using the formula: PI = (PSV − EDV)/MFV.^[15]^

### 3.2. CT brain scan

A CT brain scan was performed on admission using the TOSHIBA AQUILION PRIME 160-slice CT scanner. The scan extended from the vertex to C2, with a slice thickness of 3 mm and no contrast administration. Both sagittal and coronal reconstructions were obtained. Images were reviewed blindly by an expert radiologist using different windows (brain, subdural, and bone) to detect any trauma-related injuries. The CT findings were classified based on Marshall CT classification model and Rotterdam CT score.

### 3.3. Follow-up

Subjects were monitored for SND at day 7 post-injury through clinical observation. Neurological deterioration was determined according to one of the following predefined objective measures:

1. A reduction in the GCS by more than 2 points from the first assessment, without the use of pharmacologic sedation.
2. A significant decline in neurological condition, requiring intervention such as in-hospital mortality, mechanical ventilation, seizures, osmotherapy, admission to the ICU, or neurosurgical intervention.

Based on the presence or absence of SND, cases were classified into 2 groups:

- The neurological deterioration group.
- The non-deterioration group.

## 4. Statistical analysis

Data were analyzed using Version 27.0 of the Statistical Package for Social Sciences. Quantitative data are expressed as mean ± standard deviation or median interquartile range, as appropriate. Qualitative data are presented as frequency and percentage.

The following statistical tests were employed:

- Independent-samples *t*-test: Used for comparing the means of 2 independent groups.
- Chi-square (χ²) test: Applied to compare proportions between 2 qualitative variables.

### 4.1. Receiver operating characteristic (ROC) analysis

ROC analysis was used to evaluate the diagnostic performance of continuous variables. The ROC curve plots the true positive rate (sensitivity) against the false positive rate (1-specificity) for various cutoff values of a parameter. Each point on the ROC curve corresponds to a sensitivity/specificity pair for a specific threshold. The area under the curve (AUC) indicates the ability of a parameter to distinguish between 2 diagnostic groups (e.g., diseased vs normal). A 95% confidence interval (CI) and a 5% margin of error were used for all tests. A *P*-value < 0.05 was considered statistically significant.

## 5. Results

Fifty-five subjects were recruited for this study. All participants were admitted to the Emergency Care Unit of Helwan University Hospital over an 18-month period, from October 2021 to April 2023, and were diagnosed with TBI.

### 5.1. Demographics

The general characteristics (gender and age) of all enrolled patients, as well as those in the 2 primary outcome groups (with and without SND), assessed at day 7 post-trauma, are presented in Table 1. Nineteen patients (34.5%) developed SND, while 36 patients (65.5%) did not. Demographic characteristics, including age and sex, were comparable between the 2 groups, and no statistically significant differences were detected (*P*-value > 0.05).

**Table 1:** Comparison of demographic data between patients with and without neurological deterioration.

Variable	Total (N = 55)		With neurological deterioration (n = 19)	Without neurological deterioration (n = 36)	*P*-value
Age (yr)	31.6 ± 10.9	32.5 ± 11.0		31.2 ± 11.1	.693
Sex, n (%)					.218
Male	35 (63.6%)	10 (52.6%)		25 (69.4%)	
Female	20 (36.4%)	9 (47.4%)		11 (30.6%)	

### 5.2. Mode of trauma

**Figure 1.**
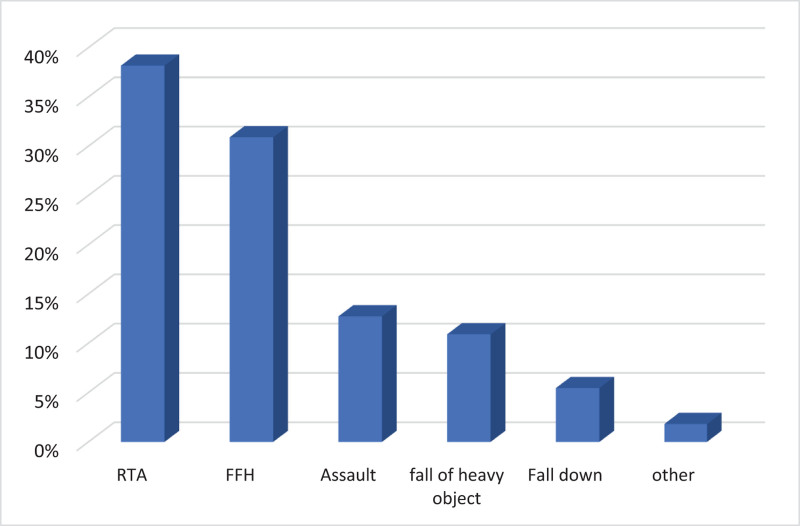
Distribution of cases according to mode of trauma. FFH = falls from height, RTA = road traffic accident.

The 2 groups were analyzed based on the mode of trauma. The predominant mode of trauma observed in the study cohort was RTA, representing for 21 cases (38%), then FFH, with 17 cases (31%). There was a statistically significant variation between the groups regarding the mode of trauma (*P*-value = 0.015, χ²). Among patients who developed SND, the most frequent mechanism of injury was RTA (12 cases, 63.2%), while among patients without SND, FFH was more common (15 cases, 41.7%) (Fig. 1).

### 5.3. CT findings

**Figure 2.**
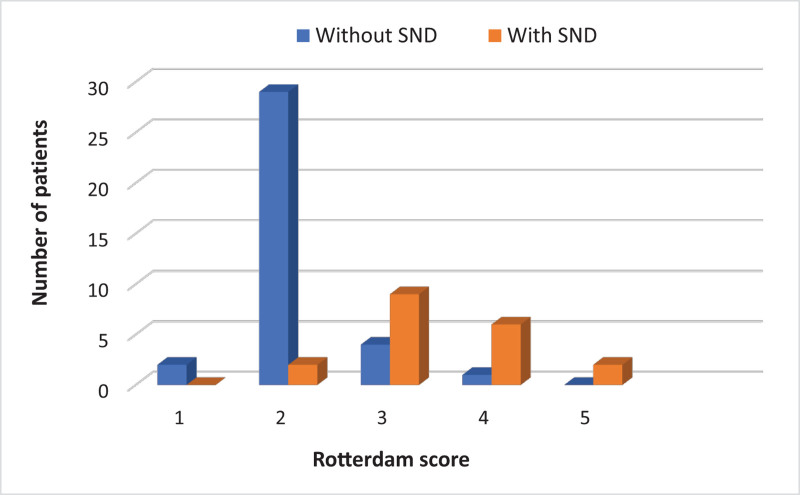
Comparison between 2 groups according to Rotterdam CT brain score. CT = computed tomography, SND = secondary neurological deterioration.

**Figure 3.**
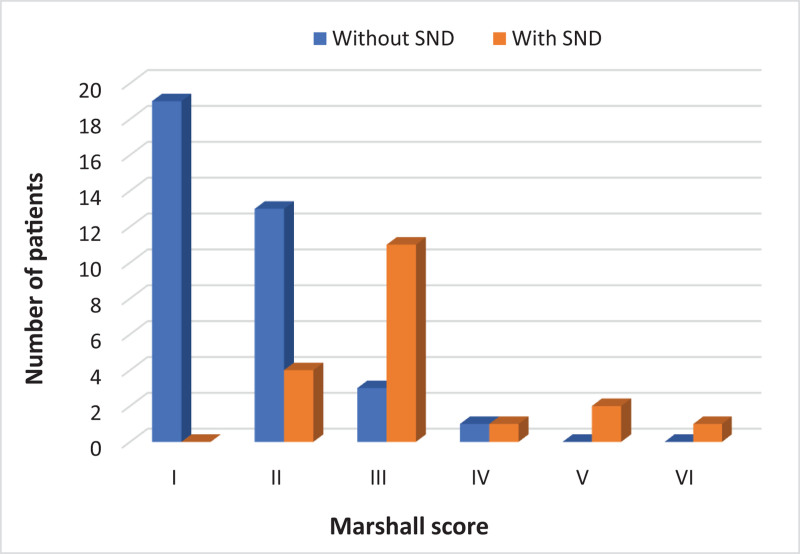
Comparison between 2 groups according to Marshall CT brain score. CT = computed tomography, SND = secondary neurological deterioration.

Comparison of the Rotterdam CT brain score on admission revealed a statistically significant difference between the 2 groups (*P* < .001), with cases who developed SND having higher Rotterdam scores (Fig. 2). In a similar manner, a statistically significant difference was detected in the Marshall CT brain score (*P* < .001), with higher scores found in patients with SND (Fig. 3).

ROC analysis was conducted to determine the optimal cutoff values for predicting neurological deterioration at day 7 based on CT-based prognostic markers. The Rotterdam CT score on admission achieved an AUC of 0.899 (95% CI: 0.813–0.985, *P* < .001), with a cutoff value of 3. This yielded a sensitivity of 89.5% (95% CI: 66.9–98.7), specificity of 86.1% (95% CI: 70.5–95.3), and accuracy of 87.3%. Similarly, the Marshall CT score on admission provided an AUC of 0.898 (95% CI: 0.822–0.974, *P* < .001) at the same cutoff value, with 78.9% sensitivity (95% CI: 54.4–93.9), 88.9% specificity (95% CI: 73.9–96.9), and 85.5% accuracy (Table 3, Fig. 7).

### 5.4. Glasgow coma scale

Among the 55 patients, 49 presented with mild to moderate TBI as indicated by a GCS score between 9 and 15, while 9 patients presented with severe TBI, with a GCS score of <9. On day 7 post-trauma, 43 patients maintained a GCS score between 9 and 15, while 12 patients had a GCS of <8.

**Figure 4.**
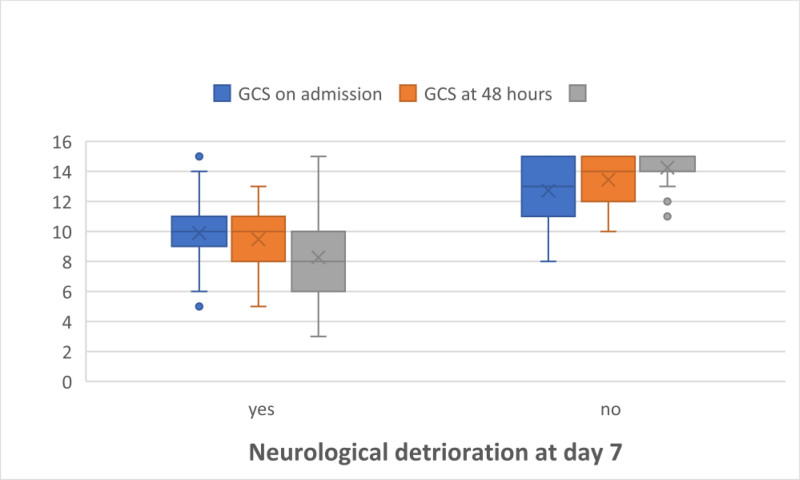
Box and whisker comparison graph between groups. GCS = Glasgow Coma Scale.

Comparison of the GCS scores (on admission, 48 hours post-trauma, and on day 7) revealed a statistically significant difference between the 2 groups (*P* < .001), with cases that developed SND having significantly lower GCS scores at admission, after 48 hours, and at day 7 (Fig. 4).

Of the 19 patients (57.9%) who developed SND by day 7, 11 patients demonstrated a decline of over 2 points in the GCS compared to their admission score. The remaining patients with SND developed additional neurological signs and symptoms, necessitating further medical or surgical intervention (Table 2).

**Table 2:** Neurological data of patients with secondary neurological deterioration.

Outcome	Category	Group with neurological deterioration (n = 19)
↓ GCS > 2 at 7 d	No	11 (57.9%)
Newly developed neurological signs requiring further intervention	Yes	9 (41.1%)

GCS = Glasgow coma scale

### 5.5. TCD measurements

**Figure 5.**
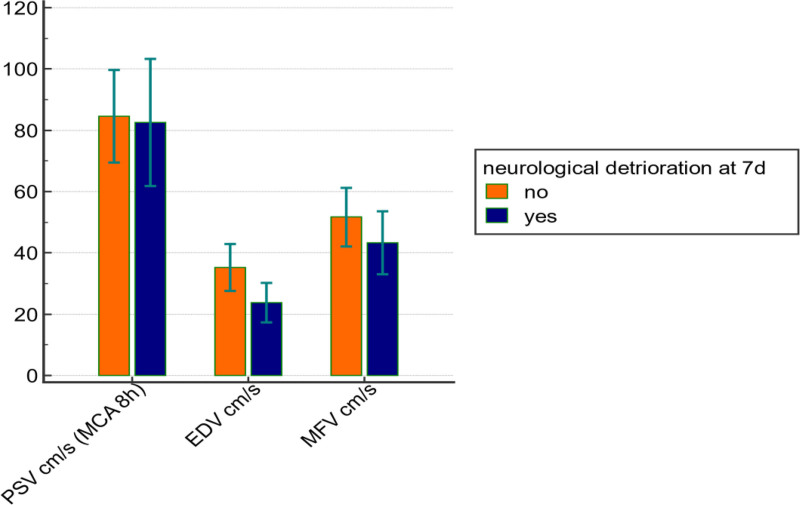
Bar comparison graph between groups as regard Doppler data. EDV = end-diastolic velocity, MFV = mean flow velocity, PSV = peak systolic velocity.

**Figure 6.**
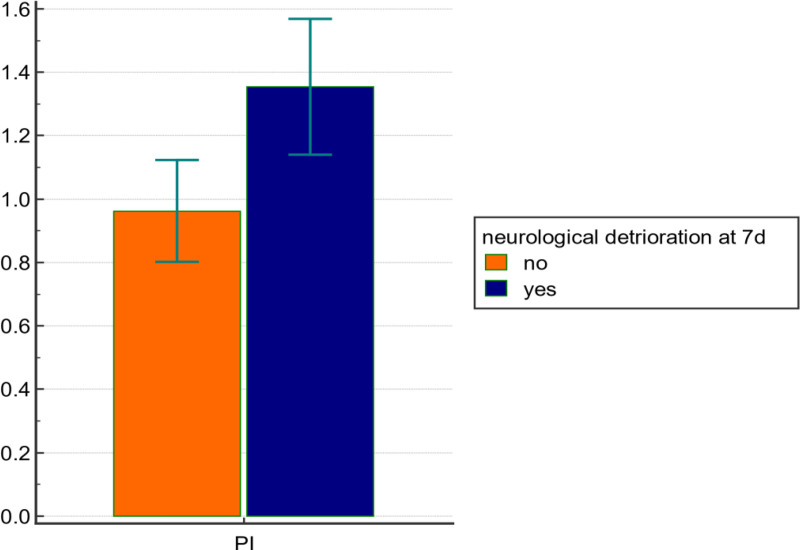
Bar comparison graph between groups as regard Doppler data. PI = pulsatility index.

Based on early TCD measurements of the MCA, performed within 8 hours post-trauma, patients who developed SND by day 7 exhibited significantly lower EDV and MFV, as well as significantly higher PI, compared to those who did not develop SND. These differences were statistically significant, with *P* < .001 for both EDV and PI, and *P* = .004 for MFV. The 2 groups showed no significant difference in PSV. (Figs. 5–6).

When both the Rotterdam and Marshall CT scores were combined, the model demonstrated a sensitivity of 89.5%, specificity of 83.3%, and accuracy of 85.5%.

**Figure 7.**
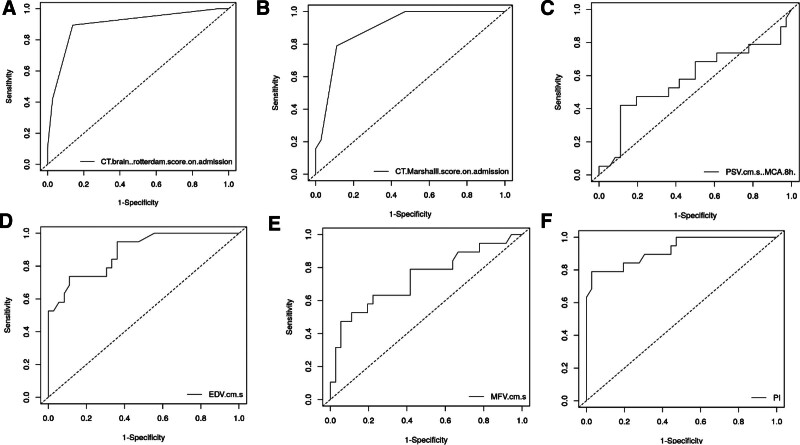
ROC curve of studied parameters. (A) CT brain Rotterdam score on admission, (B) CT Marshall score on admission, (C) PSV, (D) EDV, (E) MFV, and (F) PI. CT = computed tomography, EDV = end-diastolic velocity, MFV = mean flow velocity, PI = pulsatility index, PSV = peak systolic velocity, ROC = receiver operating characteristic.

ROC analysis was utilized to assess the diagnostic performance of TCD parameters in predicting SND at day 7 post-trauma:

- EDV demonstrated strong predictive ability, with an AUC of 0.884 (95% CI: 0.794–0.974, *P* < .001). A cutoff value of ≤ 24.9 cm/s yielded a sensitivity of 73.68% (95% CI: 48.8–90.9), specificity of 88.89% (95% CI: 73.9–96.9), and accuracy of 83.6%.
- PI showed excellent performance, with an AUC of 0.923 (95% CI: 0.843–0.999, *P* < .001). A cutoff value of > 1.299 achieved a sensitivity of 78.95% (95% CI: 54.4–93.9), specificity of 97.20% (95% CI: 85.5–99.9), and accuracy of 90.9%.
- MFV had an AUC of 0.734 (95% CI: 0.584–0.884, *P* = .002) with a cutoff value of ≤ 38.57 cm/s, yielding a sensitivity of 47.37% (95% CI: 24.4–71.1), specificity of 94.44% (95% CI: 81.3–99.3), and accuracy of 78.2% (Table 3, Fig. 7).

**Table 3:** Diagnostic performance of TCD parameters and CT scores.

Variable	AUC (95% CI)	*P* value	Cutoff	Sensitivity (95% CI)	Specificity (95% CI)	Accuracy
TCD parameters						
PSV cm/s (MCA 8h)	0.58 (0.41–0.76)	.365	≤70	42.1% (20.3–66.5)	88.9% (73.9–96.9)	72.7%
EDV cm/s	0.88 (0.79–0.97)	<.001	≤24.9	73.7% (48.8–90.9)	88.9% (73.9–96.9)	83.6%
MFV cm/s	0.73 (0.58–0.88)	.002	≤38.6	47.4% (24.4–71.1)	94.4% (81.3–99.3)	78.2%
PI	0.92 (0.84–1.00)	<.001	≥1.30	79.0% (54.4–93.9)	97.2% (85.5–99.9)	90.9%
CT Scores						
Rotterdam score (admission)	0.90 (0.81–0.99)	<.001	≥3	89.5% (66.9–98.7)	86.1% (70.5–95.3)	87.3%
Marshall score (admission)	0.90 (0.82–0.97)	<.001	≥3	78.9% (54.4–93.9)	88.9% (73.9–96.9)	85.5%

AUC = area under curve, CI = confidence interval, CT = computed tomography, EDV = end-diastolic velocity, MFV = mean flow velocity, PI = pulsatility index, PSV = peak systolic velocity, TCD = transcranial Doppler.

When EDV, MFV, and PI were combined, the predictive performance improved significantly, achieving a sensitivity of 89.5%, specificity of 86.1%, and overall accuracy of 87.3%.

### 5.6. In-hospital mortality

**Figure 8.**
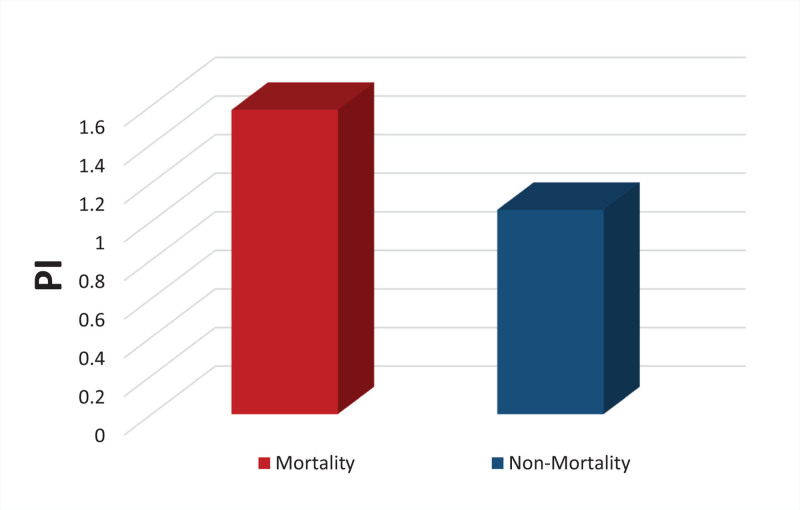
Mortality and non-mortality groups according to PI. PI = pulsatility index.

The overall mortality rate was 7.27%, with 4 patients dying within 1 month following the trauma. A comparison of Doppler indices across the mortality and non-mortality groups demonstrated statistically significant differences. The PI was significantly higher in the mortality group (mean PI = 1.57) compared to the non-mortality group (mean PI = 1.059), with *P* < .0001 (Fig. 8).

**Figure 9.**
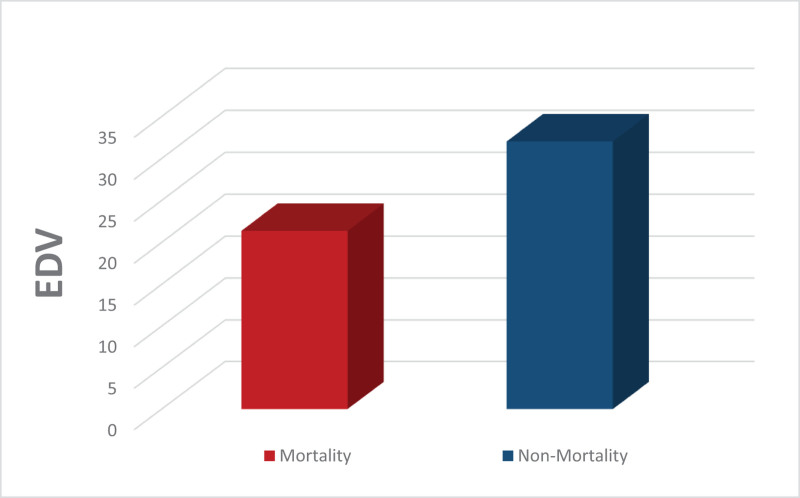
Mortality and non-mortality groups according to EDV. EDV = end-diastolic velocity.

Additionally, the EDV was significantly lower in the mortality group (mean EDV = 21.36 cm/s) compared to the non-mortality group (mean EDV = 32.02 cm/s), with a *P*-value of .022 (Figs. 8 and 9).

## 6. Discussion

TBI is a serious global healthcare and economic concern. It affects people of all ages and is common in both high- and low-income countries. The condition causes around 1.5 million deaths each year, and millions more are treated in emergency departments.^[16]^ In a substantial proportion of head trauma patients, CBF is very low and close to the ischemic level immediately following TBI. In this context, maintaining adequate CBF is critical for preventing secondary insults, which is the focus of modern TBI management. Therefore, following similar injuries, early identification and prevention of cerebral ischemia are crucial in managing this vulnerable patient population.^[17]^

All studied patients were followed for the development of SND at day 7 post-trauma and were classified into 2 groups: with and without SND. Our results showed that 36 patients (65.5%) did not develop SND, while 19 patients (34.5%) did at day 7. These findings are comparable to those of an observational prospective study by Bouzat et al,^[5]^ which reported elevated intracranial pressure in 11 patients and a decrease in GCS in 10 patients, 8 of whom required intervention.

According to TCD measurements of the MCA recorded within 8 hours of trauma in our cohort, the mean ±  standard deviation values were as follows: PSV = 83.8 ± 16.9 cm/s, EDV = 31.2 ± 9.02 cm/s, MFV = 48.7 ± 10.3 cm/s, and PI = 1.09 ± 0.22. EDV and MFV were significantly lower in patients who developed SND by day 7, with *P*-values of < .001 and .004, respectively. In contrast, PI was significantly higher in the SND group (mean = 1.35 ± 0.2) compared to the non-SND cases (mean = 0.96 ± 0.2), with a *P*-value < .001. PSV showed no statistically significant association with SND at day 7.

In a similar context, Jaffres et al^[14]^ found that patients who deteriorated had significantly higher TCD-derived PI values compared to those who did not, despite having similar injury severity. However, they reported no significant differences in PSV, EDV, or MFV across the studied groups. This discrepancy may be attributed to their inclusion of sedated, mechanically ventilated patients and/or those receiving vasopressors, all of which can influence cerebral blood flow velocities. Supporting our findings, Bouzat et al^[5]^ also reported significant differences in TCD parameters at admission between the group who developed SND and the other group who did not. Specifically, patients with SND had higher PI and lower EDV, MFV, and PSV. They identified EDV < 25 cm/s and PI ≥ 1.25 as the most accurate TCD thresholds for predicting neurological deterioration.

When assessing the AU-ROC for SND prediction using TCD measurements recorded within 8 hours after trauma, the following results were observed: AU-ROC for PI was 0.923, with a sensitivity of 78.95%, specificity of 97%, and an optimal cutoff value of > 1.23 for predicting SND at day 7 post-trauma. Similarly, EDV was identified as an independent predictor of SND, with an AU-ROC of 0.884, sensitivity of 73.68%, specificity of 88.89%, and a best cutoff value of ≤ 24.9 cm/s. While the AU-ROC for MFV was acceptable (0.734), its predictive performance was weaker compared to EDV and PI, with a sensitivity of 47.37%, specificity of 94.4%, and a best cutoff value of ≤ 38.57 cm/s. Bouzat et al^[5]^ found that cases with SND had lower PSV, MFV, and EDV, and higher PI values compared to those without SND. The reported AUCs for each parameter were 0.719 for PSV, 0.885 for MFV, 0.929 for EDV, and 0.951 for PI. The AUCs of MFV, EDV, and PI were significantly higher than that of PSV. EDV, with a cutoff < 25 cm/s, had a sensitivity of 92% and specificity of 76% for predicting SND, while PI with a cutoff ≥ 1.25 achieved 90% sensitivity and 91% specificity, as per logistic regression analysis.

In our study, the creation of a combined predictive model incorporating the significant TCD-based parameters (PI, EDV, and MFV) resulted in improved predictive performance, with a sensitivity of 89.5%, specificity of 86.1%, and overall accuracy of 87.3%.

Regarding the Marshall classification of the CT head images of our studied patients upon admission: 19 cases (34.5%) had a score of I, 17 cases (30.9%) had a score of II, 14 cases (25.4%) had a score of III, 2 cases (3.6%) had a score of IV, 2 cases (3.6%) had a score of V, and 1 case (1.8%) had a score of VI. The CT analysis revealed a statistically significant difference among the studied groups (with and without SND) in terms of Marshall Score, which was higher in the SND group.

Similarly, Jaffres et al^[14]^ reported a significant difference in the Marshall Classification between cases who developed SND and those who did not.

Based on the Rotterdam score on admission, patients who developed SND had higher scores compared to those who did not. This agrees with the findings of Taylor et al^[2]^, who conducted a study on 150 TBI patients monitored for outcomes over 2 weeks and found that the Rotterdam score was significantly correlated with patient prognosis.

The combined predictive performance of the 2 CT-based prognostic scores (Marshall and Rotterdam) for predicting SND at day 7 post-trauma showed a sensitivity of 89.5%, specificity of 83.3%, and an overall accuracy of 85.5%.

When comparing the combined predictive performance of the CT scores to that of the previously described TCD parameters, the results appear closely matched. Both methods demonstrate equal sensitivity, with the CT scores showing slightly higher specificity, while the TCD parameters exhibit slightly higher overall accuracy. Additionally, when comparing the most sensitive Doppler index, PI, alone to the predictive performance of the CT scores, we found that PI alone has higher sensitivity and specificity. Moreover, when additional practical factors are considered, such as easier accessibility, greater availability, reduced need for patient transportation, and absence of radiation exposure, TCD emerges as a favorable tool for outcome prediction in TBI cases.

In view of our findings, EDV, MFV, and PI are good predictors of early neurological deterioration (within 7 days) after TBI. However, variations in predictive parameters and thresholds reported by different authors, including our results, can be explained by differences in the definitions of SND, sample sizes, inclusion and exclusion criteria, timing of TCD parameter measurements, and whether cutoff values or trends in TCD parameters were used.

Regarding the role of TCD values in predicting mortality, our results demonstrated no significant association between measured PSV and MFV and in-hospital mortality. However, both the PI and EDV were found to be significant predictors, with significantly higher PI and lower EDV detected in the non-survivor group. In the same context, Abdalla et al^[18]^ found that PSV, EDV, and MFV were significantly higher on days 2 to 4 in survivors compared to non-survivors. They concluded that TCD-derived parameters could predict survival in the TBI cases studied.

The crucial finding of this research is that early variations in cerebral perfusion following TBI can serve as a predictor of patient outcomes.

Tracings of the MCA were recorded over a period of ten or more cardiac cycles; however, these intermittent TCD measurements could potentially miss significant peaks or troughs due to moment-to-moment variations in velocity. Therefore, continuous TCD monitoring (using a probe headset) during the early hours after admission may provide greater accuracy and yield additional valuable information.

Evaluating the outcomes of the studied cases over a longer period (e.g., 1 month or 6 months post-injury), in addition to their status at 1 week, would be beneficial for correlating early TCD measurements, CT scores, and initial clinical assessments with long-term prognosis.

The limitations of the current research also include the relatively small, single-center study population. Future studies with larger sample sizes and multiple centers would improve the external validity of our findings.

## 7. Conclusion

A considerable proportion of TBI patients may develop SND within the first week after injury. TCD conducted soon after admission may aid in detecting patients at risk of neurological decline. Thus, it may serve as a valuable measure for hospital triage, determining the appropriate level and timing of monitoring, and guiding discharge decisions. MFV, EDV, and PI obtained within the first 8 hours post-trauma may be effective predictors of SND at 1 week. Additionally, PI and EDV may serve as reliable predictors of in-hospital mortality. Therefore, we recommend future studies with larger sample sizes and multicenter studies to validate these findings.

## Author contributions

**Conceptualization:** Mona Gamalludin Alkaphoury.

**Data curation:** Ahmed Mohamed Ekbal Ghoneim, Mona Gamalludin Alkaphoury.

**Formal analysis:** Ahmed Mohamed Ekbal Ghoneim.

**Investigation:** Ahmed Mohamed Ekbal Ghoneim.

**Methodology:** Ahmed Mohamed Ekbal Ghoneim, Waleed Abdelhamid Hetta.

**Resources:** Mona Gamalludin Alkaphoury.

**Software:** Ahmed Mohamed Ekbal Ghoneim, Mona Gamalludin Alkaphoury.

**Supervision:** Safaa Kamal Mohamed, Waleed Abdelhamid Hetta, Mona Gamalludin Alkaphoury.

**Writing – original draft:** Ahmed Mohamed Ekbal Ghoneim.

**Writing – review & editing:** Safaa Kamal Mohamed, Waleed Abdelhamid Hetta, Mona Gamalludin Alkaphoury.
